# Differential Neurovirulence of African and Asian Genotype Zika Virus Isolates in Outbred Immunocompetent Mice

**DOI:** 10.4269/ajtmh.17-0263

**Published:** 2017-08-14

**Authors:** Nisha K. Duggal, Jana M. Ritter, Erin M. McDonald, Hannah Romo, Farshad Guirakhoo, Brent S. Davis, Gwong-Jen J. Chang, Aaron C. Brault

**Affiliations:** 1 Division of Vector-Borne Diseases, Centers for Disease Control and Prevention, Fort Collins, Colorado;; 2 Infectious Diseases Pathology Branch, Division of High Consequence Pathogens and Pathology, Centers for Disease Control and Prevention, Atlanta, Georgia;; 3 GeoVax, Inc., Smyrna, Georgia

## Abstract

Although first isolated almost 70 years ago, Zika virus (ZIKV; *Flavivirus, Flaviviridae*) has only recently been associated with significant outbreaks of disease in humans. Several severe ZIKV disease manifestations have also been recently documented, including fetal malformations, such as microcephaly, and Guillain–Barré syndrome in adults. Although principally transmitted by mosquitoes, sexual transmission of ZIKV has been documented. Recent publications of several interferon receptor knockout mouse models have demonstrated ZIKV-induced disease. Herein, outbred immunocompetent CD-1/ICR adult mice were assessed for susceptibility to disease by intracranial (i.c.) and intraperitoneal (i.p.) inoculation with the Ugandan prototype strain (MR766; African genotype), a low-passage Senegalese strain (DakAr41524; African genotype) and a recent ZIKV strain isolated from a traveler infected in Puerto Rico (PRVABC59; Asian genotype). Morbidity was not observed in mice inoculated by the i.p. route with either MR766 or PRVABC59 for doses up to 6 log_10_ PFU. In contrast, CD-1/ICR mice inoculated i.c. with the MR766 ZIKV strain exhibited an 80–100% mortality rate that was age independent. The DakAr41524 strain delivered by the i.c route caused 30% mortality, and the Puerto Rican ZIKV strain failed to elicit mortality but did induce a serum neutralizing immune response in 60% of mice. These data provide a potential animal model for assessing neurovirulence determinants of different ZIKV strains as well as a potential immunocompetent challenge model for assessing protective efficacy of vaccine candidates.

## INTRODUCTION

Zika virus (ZIKV) is an emerging flavivirus that has recently expanded its geographic range across the south Pacific^1^ and the Americas.^2^ Concurrent with this geographic expansion into areas without previously documented ZIKV transmission has been the link of ZIKV transmission with severe in utero fetal malformations^3,4^ and Guillain–Barré syndrome.^5–7^ The current outbreak in the western hemisphere has also highlighted the potential for sexual transmission in addition to mosquito transmission. These factors have fueled significant ongoing efforts in vaccine development to reduce the disease burden in human populations.

A requisite need for the development of vaccines and vaccination regimens is the development of applicable animal models for testing immunogenicity and the assessment of protective efficacy. Recent reports have described the development of mouse models deficient in interferon responses that demonstrated significant viremias, viral tissue loads, and mortality after inoculation with various ZIKV strains.^8–10^ Although these models have demonstrated the elicitation of morbidity and mortality after experimental inoculation with low-passage viruses administered by subcutaneous, intraperitoneal (i.p.), intradermal, intravaginal, and intravenous routes, the lack of an intact interferon response precludes the assessment of the role of cell-mediated immune responses on protective efficacy of replication competent vaccine candidates. Previous inoculation of immunocompetent mice by i.p. inoculation failed to elicit any viremia or observable sign of morbidity after inoculation of low-passage Asian genotype ZIKV strains^8^; however, intravenous inoculation of Balb/c mice with very high viral doses was found to lead to increased ZIKV RNA levels after inoculation.^11^ Intracranial (i.c.) inoculation of 35-day-old immunocompetent mice with the MR766 prototype ZIKV isolate, which had been passaged extensively in suckling mouse brains, has previously demonstrated signs of morbidity within 5–15 days post inoculation (dpi).^12^ In this report, immunocompetent mice (CD-1/ICR) were assessed for susceptibility to both i.p. and i.c. inoculation routes with both African and Asian ZIKV genotype viruses. These data provide an applicable model for assessing immunogenicity and protective efficacy in an immunocompetent mouse model as well for the direct interrogation of ZIKV viral genetic determinants associated with differential neurovirulence phenotypes.

## METHODS

### Viruses.

Representative African (MR766 and DakAr41524) and Asian genotype (PRVABC59) ZIKV strains were used. The MR766 virus was the prototype isolate from Uganda in 1947 and had been passaged up to 149 times in suckling mouse brain and twice in Vero cell culture. In contrast, the DakAr41524 and PRVABC59 viruses were low-passage strains (Vero passage 7 and 3, respectively) originally isolated in 1984 and 2015, respectively. To confirm the sequence of the virus stocks, RNA was extracted using the Qiagen Viral RNA mini kit, and the viral genomes were amplified by one-step RT-PCR (Qiagen) using specific primers to produce seven overlapping RT-PCR products. RT-PCR products were sequenced directly. Complete termini sequences were obtained from 5′ and 3′ RACE products (Invitrogen). Primer sequences are available upon request.

### Cell lines.

Green monkey kidney fibroblast (Vero) and human neuroblastoma (SH-SY5Y) cells were maintained at 37°C with 5% CO_2_. Vero cells were grown in DMEM supplemented with 10% FBS and 1% penicillin–streptomycin. The SH-SY5Y cell line was a gift from D. Beckham (University of Colorado) and was maintained in DMEM/F12 supplemented with 10% FBS and 1% penicillin–streptomycin. For differentiation, SH-SY5Y cells were plated in six-well plates coated with poly-l-lysine at 1 × 10^5^ cells/well, and differentiation was performed according to a previous report.^13^ Briefly, 2 days after plating, freshly prepared 10 µM all-trans retinoic acid (RA; Sigma-Aldrich) was added to the media. Three days later, the media was changed to Neurobasal-A medium minus phenol red supplemented with 1% l-glutamine, 1% penicillin–streptomycin, 1% N-2 supplement (ThermoFisher); and 50 ng/mL human BDNF (Sigma Aldrich) and maintained for 3 days before viral inoculation. Cells were inoculated in triplicate at a multiplicity of infection of 0.1 for the two viruses. Culture supernatant was sampled daily from 1 to 7 dpi and assayed for ZIKV titer by plaque assay on Vero cells. Statistical significance of differences in mean titer was determined by performing multiple *t* tests with a Holm–Sidak correction for multiple comparisons in Prism7.

### Mouse inoculations.

Groups of five 4-week-old (wko) female CD-1/ICR mice (Charles River) were inoculated by i.p. inoculation with a 10-fold dilution dose series of MR766 or PRVABC59 virus strains [range: 6 log_10_–1 log_10_ plaque forming units (PFU)]. Mice were observed daily for any signs of disease. Additional groups of ten 4-, 6-, 8-, and 12-wko female CD-1/ICR mice received i.c. inoculations after isoflurane anesthesia in the right brain hemisphere with a 30-gauge needle affixed to a Hamilton syringe sheathed by a pipette tip allowing no more than a 4-mm needle penetrance into the skull cavity. For the i.c. inoculations, 5 log_10_ PFU of MR766, DakAr41524, or PRVABC59 diluted in phosphate buffered saline (PBS) solution or PBS alone were administered in a 10 µL inoculum. Inoculated mice were placed back in their cages and monitored for recovery from anesthesia. Three additional groups of ten 4-wko CD-1/ICR mice were inoculated i.c. with 5 log_10_ PFU of MR766, DakAr41524, or PRVABC59 as described previously, and three to four mice were euthanized from both inoculation groups on dpi 1, 3, and 5. Three additional mice were similarly inoculated i.c. with PBS to serve as histological controls. All inoculated mice were monitored twice daily until clinical signs of morbidity were observed, at which point monitoring was increased to four times daily. Mice were weighed once per day from the day of inoculation to the termination of the study at dpi 24. Any mouse that lost ≥ 15% body weight or showed signs of encephalitis (e.g., incoordination, ataxia, limb weakness/paralysis) was euthanized by isoflurane anesthesia followed by cervical dislocation. All animal studies were conducted under approved IACUC protocols at the Centers for Disease Control and Prevention.

### ZIKV infectious titer assessment.

Mice meeting euthanasia criteria or at aforementioned sampling time points were subjected to deep isoflurane anesthesia and bled by cardiac puncture before cervical dislocation. After euthanasia, the brains of inoculated mice were removed and one hemisphere collected for viral titration and the other hemisphere fixed in 10% neutral buffered formalin for histology. Viral titers from the brains were determined by homogenizing brain tissue in BA-1 media using a pestle, clarifying by centrifugation, and plaque titrating on Vero cells as described previously.^14^ Sera from CD-1/ICR mice inoculated i.c. were also assessed for viremia by Vero cell plaque assay. Statistical significance of differences in mean titer was determined by performing multiple *t* tests with a Holm–Sidak correction for multiple comparisons in Prism7.

### Neutralizing antibody titrations (PRNT_90_).

At 24 dpi, surviving mice were anesthetized with inhalational isoflurane, and approximately 0.5 mL of whole blood was obtained by cardiac puncture at which point the mice were euthanized by cervical dislocation. Whole blood was collected in serum separator tubes and spun at 3,500 × *g* for 5 minutes. A portion of the serum was assessed for infectious virus as described previously, and the remaining serum was heat-inactivated at 56°C for 30 minutes, serially diluted 2-fold, and incubated with approximately 100 PFU MR766 or PRVABC59 for 1 hour at 37°C. The samples were titrated as described for plaque assays, and neutralization activity was identified at a 90% plaque reduction threshold as compared with serum negative controls. The lowest serum dilution tested for neutralization of PRVABC59 was 1:20, and the lowest serum dilution tested for neutralization of MR766 was 1:40 because of limited volume of sample. Statistical significance of the differences in proportions of mice with neutralizing antibody responses was determined by Fisher’s exact test. Statistical significance of differences in mean PRNT_90_ titer between virus groups was determined by performing a *t* test in Prism7.

### Histology and immunohistochemistry.

Tissues were fixed in 10% neutral buffered formalin for 3 days and then transferred to 70% ethanol for storage until processed for routine paraffin histology. Sections were cut at 4 μm and stained with hematoxylin and eosin or by immunohistochemical assay (IHC) for ZIKV antigen before evaluation. The IHC assay was performed using a polymer-based indirect immunoalkaline phosphatase detection system with colorimetric detection of antibody/polymer complex with Fast Red Chromogen. The primary antibody used was a rabbit polyclonal antibody generated against ZIKV VLPs.^15^ Appropriate positive and negative controls were performed in parallel.

## RESULTS

### Differential in vitro replication of ZIKV strains in neuronal cells.

**Figure 1.**
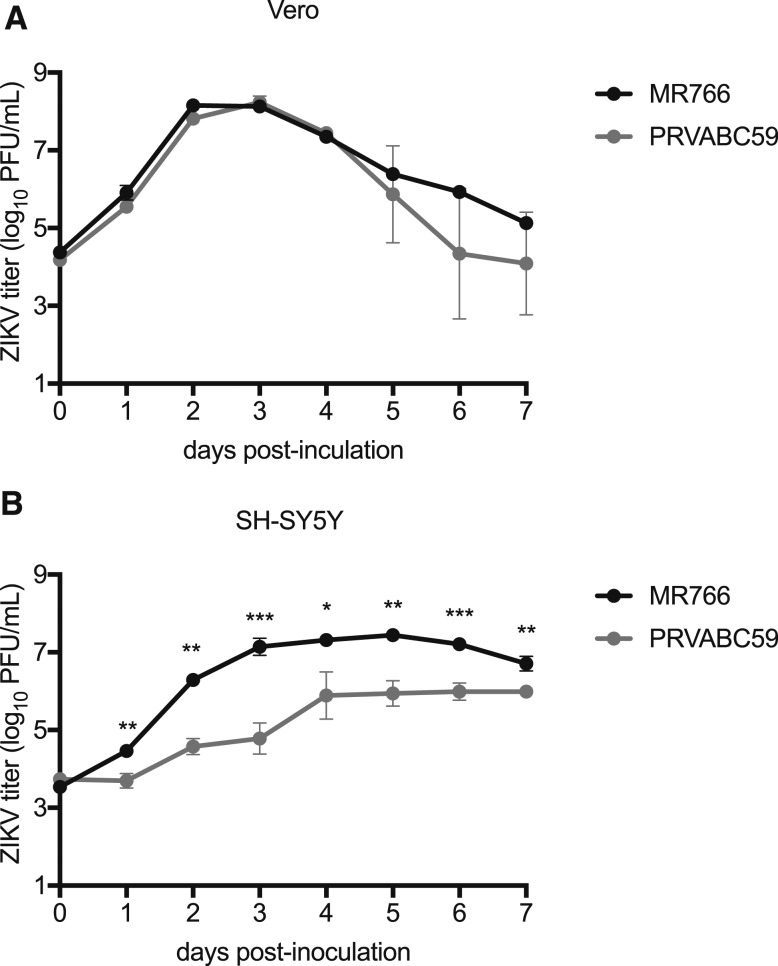
Growth of MR766 (dark circles) and PRVABC59 (gray circles) ZIKV isolates in Vero cells (**A**) and differentiated SH-SY5Y human neuroblastoma cells (**B**). Circles and error bars represent means and standard deviations, respectively, of triplicate inoculated cultures. Growth curves experiments were performed three times, with one representative experiment shown. *P < 0.05, **P < 0.01, ***P < 0.001.

The sequence differences between MR766 and PRVABC59 that are unique to MR766 within the African genotype are shown in Table 1. These seven amino acid differences represent potential adaptive mutations acquired through extensive mouse brain passaging. To assess the potential neuronal adaptation of MR766 during its serial i.c. passage, the in vitro replication kinetics of MR766 and PRVABC59 were assessed in differentiated SH-SY5Y cells (human neuroblastoma) and monkey kidney fibroblast (Vero) cells. MR766 and PRVABC59 grew similarly in Vero cells, with no statistically significant differences in peak viral titer (8.15 and 8.24 log_10_ PFU/mL, respectively, *P* = 0.36; Figure 1A). In contrast, MR766 manifested significantly higher titers than PRVABC59 in differentiated human neuronal SH-SY5Y cells at all time points, with peak titers of 7.45 and 5.99, respectively (*P* < 0.01, Figure 1B). At dpi 3, the mean titer of MR766 was approximately 500-fold higher than PRVABC59.

**Table 1:** Amino acid differences unique to ZIKV strain MR766

Viral protein	Amino acid	MR766	PRVABC59
E	158	Y	H
	283	K	R
	341	I	V
	343	V	A
NS1	146	E	K
NS2A	153	P	A
	180	I	F

### Inoculation of immunocompetent mice with ZIKV.

**Figure 2.**
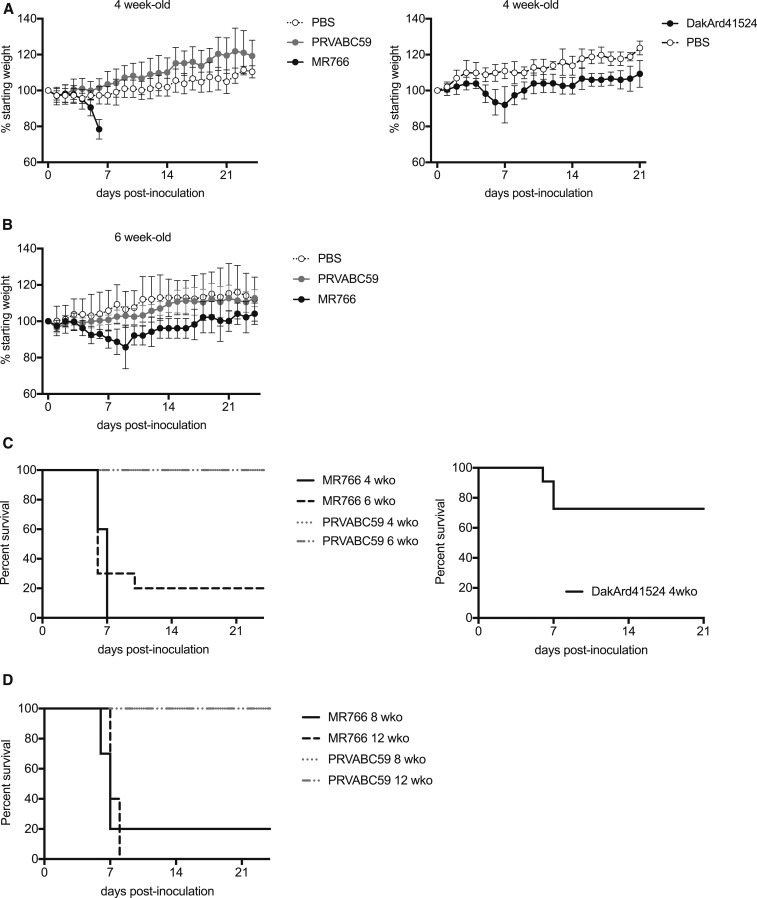
Intracranial inoculation of CD-1/ICR mice with Asian (PRVABC59) and African (DakAr41524 and MR766) ZIKV strains. (**A**) Weights (as a proportion to their initial preinoculation measurement) of 4-wko CD-1/ICR mice after i.c. inoculation. Left panel: PBS (open circles, *N* = 5), PRVABC59 (gray circles, *N* = 10), or MR766 (dark circles, *N* = 10). Right panel: PBS (open circles, *N* = 5) or DakAr41524 (dark circles, *N* = 11). Circles and error bars represent means and standard deviations, respectively. (**B**) Weights (as a proportion to their initial preinoculation measurement) of 6-wko mice after inoculation with PBS (open circles, *N* = 5), PRVABC59 (gray circles, *N* = 10), or MR766 (dark circles, *N* = 10). Two surviving mice in the 6-wko MR766 inoculation group from dpi 10–24 are representative of the increased weights over time. Circles and error bars represent means and standard deviations, respectively. (**C**) Kaplan–Meier survivorship plot for 4-wko (solid line) and 6-wko (dashed line) mice. Left panel: Mice inoculated with PRVABC59 (gray line) or MR766 (dark line) ZIKV isolates. Right panel: Mice inoculated with DakAr41524 (dark line). (**D**) Kaplan–Meier survivorship plot for 8-wko (solid line) and 12-wko (dashed line) mice inoculated with PRVABC59 (gray line, *N* = 10) or MR766 (dark line, *N* = 10) ZIKV isolates.

Of the sixty 4-wko CD-1/ICR mice inoculated intraperitoneally with doses of MR766 and PRVABC59 ranging from 6 log_10_ PFU to 1 log_10_ PFU, none demonstrated any signs of morbidity or mortality (data not shown). In contrast, mice from the four age groups (4, 6, 8, and 12 wko) inoculated i.c. with 5 log_10_ PFU MR766, but not 5 log_10_ PFU PRVABC59 or PBS, developed encephalitis and/or displayed significant weight loss (Figure 2A and B). Ninety percent (36/40) of mice inoculated by the i.c. route with MR766 developed symptoms of encephalitis that included recumbency, hunched posture, limb paralysis, ataxia, and incoordination. Mice in the four age groups had a median survivorship of 6–7 days (Figure 2C and D). In contrast, 100% of mice inoculated i.c. with PRVABC59 (40/40) or PBS (10/10) survived to the end of the study (Figure 2C and D). In a separate experiment, 4-wko CD-1/ICR mice were inoculated i.c. with DakAr41524 or PBS, and 30% (3 out of 11) inoculated with DakAr41524 developed symptoms of encephalitis and weight loss, whereas none of the PBS controls did (Figure 2A and C).

**Figure 3.**
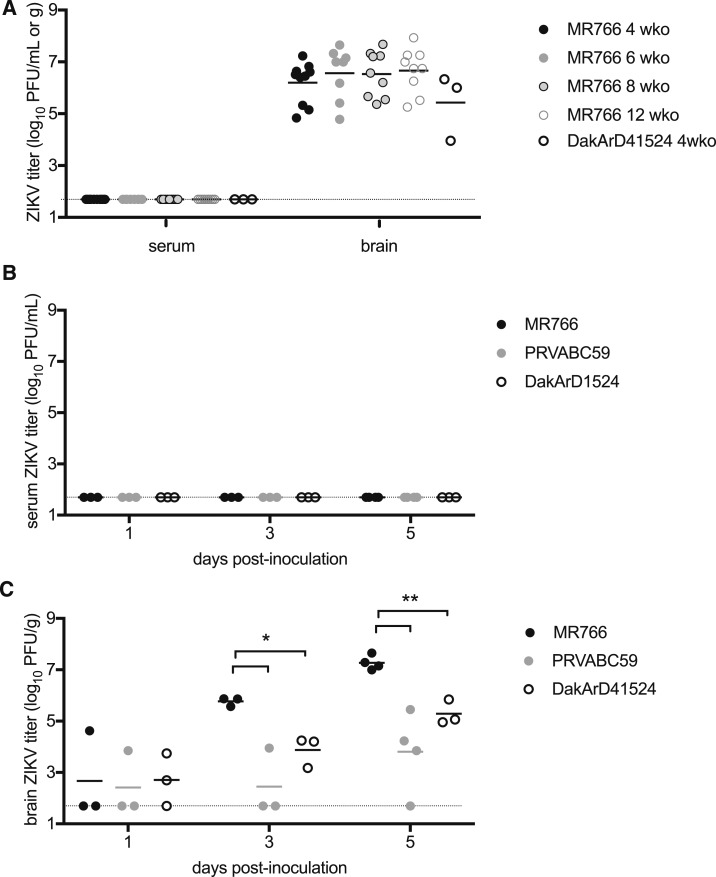
Serum and brain viral titers of CD-1/ICR mice inoculated i.c. with ZIKV. (**A**) Viremia and brain titers at the time of euthanasia in 4-wko (dark filled circles, *N* = 10), 6-wko (light filled circles, *N* = 8), 8-wko (light-filled circles with dark lines, *N* = 9), and 12-wko (open circles with light lines, *N* = 8) mice inoculated with MR766 or 4-wko mice inoculated with DakAr41524 (open circles with dark lines, *N* = 3). (**B**) Viremia of 4-wko mice on days 1, 3, and 5 after inoculation with MR766 (dark circles, *N* = 3), DakAr41524 (open circles, *N* = 3), or PRVABC59 (light circles, *N* = 3). (**C**) Brain titers of 4-wko mice on days 1, 3, and 5 after inoculation with MR766 (dark circles, *N* = 3), DakAr41524 (open circles, *N* = 3), or PRVABC59 (light circles, *N* = 3). The limit of detection is shown as a dashed line. *P < 0.05, **P < 0.01.

Serum and brain titers were determined for all mice that succumbed to MR766 or DakAr41524 i.c. inoculation. Whereas all serum samples were negative for evidence of ZIKV infectious virus (PFU), mean brain titers ranged between 5.4 and 6.7 log_10_ ZIKV PFU/g tissue with no statistically significant differences between the age groups or virus strains (Figure 3A). To evaluate early events in infection, two groups of 10 additional 4-wko CD-1/ICR female mice were inoculated i.c. with 10,000 PFU of MR766, DakAr41524, or PRVABC59 and euthanized in groups of 3 or 4 on dpi 1, 3, or 5. All serum samples were negative for evidence of ZIKV infectious units (Figure 3B), but increasing titers of infectious ZIKV were detected in the brain tissue of mice inoculated with both viruses over time (Figure 3C). Mice inoculated i.c. with MR766 had significantly higher brain titers than mice inoculated with DakAr41524 or PRVABC59 on days 3 and 5 after inoculation (*P* < 0.05 and *P* < 0.01, respectively).

### Neutralizing immune responses.

To assess ZIKV neutralizing antibody responses, PRNT_90_ assays were performed on the sera from all surviving mice inoculated i.c. Three of the four (75%) MR766 surviving i.c.-inoculated mice exhibited neutralizing responses against PRVABC59 (Table 2). Of the mice inoculated i.c. with PRVABC59, 24 of 40 (60%) exhibited neutralizing antibody responses against PRVABC59 (Table 2), which was not statistically different than MR766. In addition, there was no statistical difference between the proportions of 4-, 6-, and 12-wko mice demonstrating neutralizing responses inoculated with PRVABC59 (40–50%), with 8-wko mice demonstrating a higher proportion of neutralizing responses (100%; *P* < 0.05). The differences in the magnitude of the neutralizing response between mice inoculated i.c. with MR766 or PRVABC59 were not significantly different. Samples from the 8- and 12-wko mice were also tested for neutralizing responses to MR766. Eleven samples had detectable PRNT_90_ titers against both MR766 and PRVABC59 and were within 2-fold of each other (Table 2). One mouse inoculated with PRVABC59 had a 4-fold higher PRNT_90_ titer against PRVABC59 than MR766.

**Table 2:** Neutralizing antibody responses of surviving mice inoculated i.c. with ZIKV

Inoculating virus	Age (wko)	Mouse number	PRNT_90_ PRVABC59	PRNT_90_ MR766
MR766	8	1	320	320
MR766	8	2	80	80
PRVABC59	12	1	40	40
PRVABC59	12	2	160	320
PRVABC59	12	3	< 20	< 40
PRVABC59	12	4	< 20	< 40
PRVABC59	12	5	< 20	< 40
PRVABC59	12	6	40	< 40
PRVABC59	12	7	< 20	< 40
PRVABC59	12	8	80	< 40
PRVABC59	12	9	80	80
PRVABC59	12	10	< 20	< 40
PRVABC59	8	1	40	< 40
PRVABC59	8	2	80	40
PRVABC59	8	3	20	< 40
PRVABC59	8	4	640	160
PRVABC59	8	5	20	40
PRVABC59	8	6	160	320
PRVABC59	8	7	20	40
PRVABC59	8	8	80	160
PRVABC59	8	9	320	160
PRVABC59	8	10	20	< 40
MR766	6	1	< 20	n.d.
MR766	6	4	160	n.d.
PRVABC59	6	1	< 20	n.d.
PRVABC59	6	2	40	n.d.
PRVABC59	6	3	< 20	n.d.
PRVABC59	6	4	40	n.d.
PRVABC59	6	5	< 20	n.d.
PRVABC59	6	6	< 20	n.d.
PRVABC59	6	7	20	n.d.
PRVABC59	6	8	< 20	n.d.
PRVABC59	6	9	< 20	n.d.
PRVABC59	6	10	80	n.d.
PRVABC59	4	1	< 20	n.d.
PRVABC59	4	2	< 20	n.d.
PRVABC59	4	3	< 20	n.d.
PRVABC59	4	4	< 20	n.d.
PRVABC59	4	5	40	n.d.
PRVABC59	4	6	40	n.d.
PRVABC59	4	7	20	n.d.
PRVABC59	4	8	40	n.d.
PRVABC59	4	9	< 20	n.d.
PRVABC59	4	10	40	n.d.

n.d. = not done.

### Pathological findings.

**Figure 4.**
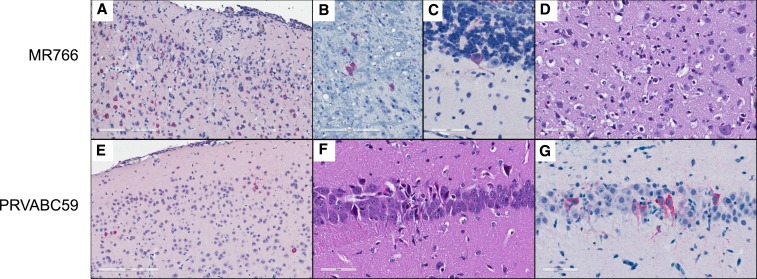
Histopathology and immunohistochemistry of brains from CD-1/ICR mice inoculated i.c. with ZIKV. (**A**) MR766-inoculated mouse brain at 5 dpi. Extensive inflammation and neuronal cell death with ZIKV labeling. ZIKV IHC, 200X. (**B**) MR766-inoculated mouse brain at 5 dpi. ZIKV labeling in scattered pontine nuclei without inflammation. ZIKV IHC, 400×. (**C**) MR766-inoculated mouse brain at 5 dpi. ZIKV labeling in a single cerebellar Purkinje cell without inflammation. ZIKV IHC, 400×. (**D**) MR766-inoculated mouse brain at 6 dpi. Many necrotic neurons within the cerebral cortex. H&E, 400×. (**E**) PRVABC59-inoculated mouse brain at 5 dpi. Scattered neuronal IHC labeling for ZIKV, without morphologic alterations or inflammation. ZIKV IHC, 200×. (**F**) PRVABC59-inoculated mouse brain at 5 dpi. Focal necrosis of hippocampal neurons. H&E, 400×. (**G**) PRVABC59-inoculated mouse brain at 5 dpi. ZIKV IHC labeling of neurons in region of hippocampal necrosis. ZIKV IHC, 400×. This figure appears in color at www.ajtmh.org.

Histopathological evaluation of brain tissue sections revealed no evidence of encephalitis or neural damage in mice inoculated i.c with either ZIKV strain at dpi 1 and 3; however, significant pathological alterations with pronounced differences between the strains were evident by dpi 5. At that point, brains from all MR766 inoculated mice exhibited widespread inflammatory and reactive changes with evidence of neuronal cell death. The cerebral cortex was most severely affected, with mixed leptomeningeal and parenchymal inflammation, glial proliferation and activation, and scattered neuronal necrosis. These changes correlated with extensive ZIKV IHC labeling throughout the cortex, predominantly in neurons (Figure 4A). Basal forebrain, hippocampus, thalamus, and brainstem showed variable degrees of similar changes, and also showed neuronal IHC labeling without morphologic evidence of cell damage or inflammatory reaction (Figure 4B). Two mice also showed rare IHC labeling in Purkinje cells of the cerebellum (Figure 4C); no MR766-inoculated mice had retinal morphologic changes or IHC labeling. Brains from nine 4-wko MR766-inoculated mice euthanized at 6 or 7 dpi showed similar but more intense and widespread neuronal cell death (Figure 4D), inflammatory reaction, and ZIKV labeling by IHC.

In contrast, mice inoculated i.c. with PRVABC59 at 5 dpi demonstrated limited brain pathology. The brain of one mouse had no discernible morphological alterations and no IHC labeling; one mouse exhibited scattered cerebral cortical neuronal labeling by IHC without overt neuronal damage (Figure 4E), and two of four had focal neuronal necrosis in the hippocampus, with localization of ZIKV antigen restricted to these areas by IHC (Figure 4F and G). No histopathologic changes or IHC labeling of ZIKV was observed in the brain outside the cortex and hippocampus, or in the retinas of the PRVABC59-inoculated mice.

The brains of four MR766-inoculated and 10 PRVABC59-inoculated mice surviving to dpi 24 were histopathologically unremarkable and showed no ZIKV labeling by IHC, except for the brain of one PRVABC59-inoculated mouse, the section from which showed IHC labeling in a region with pathological changes suggestive of the initial inoculation site.

## DISCUSSION

The results presented here indicate the potential utility of performing i.c. inoculation of immunocompetent outbred mice with the neuroadapted ZIKV strain, MR766, for initial efficacy, vaccine-induced immunogenicity, and protective efficacy assessments. ZIKV strain MR766 has been extensively adapted by serial i.c. passage in suckling mice. Immunocompetent CD-1/ICR mice were more susceptible to i.c. inoculation with MR766 than with DakArd41524 or PRVABC59, both low-passage isolates (Figure 2), yet CD-1/ICR mice were also more susceptible to i.c. inoculation with DakArd41524 (African genotype) than PRVABC59 (Asian genotype) MR766 elicited more widespread inflammation and neuronal cell death in the brains of mice inoculated i.c. than was observed in mice inoculated i.c. with PRVABC59 (Figures 3 and 4). This corresponded with increased viral loads observed in the brains in 4-, 6-, 8-, and 12-wko mice. The age independence of this phenotype could indicate the applicability of this approach as a suitable challenge model for prime-boost assessments in more aged mice that will be necessary for vaccine efficacy assessments. In other models for ZIKV challenge in which peripheral challenge of virus has been assessed through subcutaneous,^8^ i.p.,^8^ intravenous,^11^ or intrauterine/intravaginal routes,^16,17^ immunocompetent mice were not highly susceptible to establishing ZIKV infections or demonstrating significant disease; thus, previously reported mouse models for ZIKV pathogenesis have largely used immunodeficient mice,^8,9,15,18,19^ immunocompetent mice treated with antibodies targeting interferon receptors,^20–22^ or murine-adapted ZIKV strains.^21^

MR766 also grew to higher titers than PRVABC59 in a human neuroblastoma cell line while showing indistinguishable growth in a non-neuronal (Vero) cell line (Figure 1), suggesting a specific adaptation to neuronal cells that could include adaptation to neuronal-specific receptor(s). Although there are 106 amino acid differences between MR766 and PRVABC59, most of the differences are shared across African strains with low-passage history, including DakAr41524.^23^ The mutations accumulated in the MR766 isolate presumably during mouse adaptation are likely to be the seven amino acid differences that are specific to MR766, which are clustered in E, NS1, and NS2A (Table 1). Despite the extensive passaging of the MR766 isolate, these mutations do not demonstrate positive charge substitutions on the surface of the envelope protein that are characteristic of cell culture adaptation for heparin sulfate moieties in which in vivo virulence in mice is typically reduced significantly.^24^ These seven amino acid mutations could be targeted for reverse genetic studies to identify possible neurovirulence determinants of the MR766 strain and thus be incorporated into Asian-specific genetic backbones^14,25,26^ to serve as more representative challenge strains for the current ZIKV epidemic for vaccine studies. Nevertheless, the finding of no significant difference in the proportion of surviving mice that developed neutralizing titers between the two viruses indicates that potential mouse-adapted mutations do not appear to significantly negatively impact immunogenicity. In addition, the immune response of the surviving mice inoculated i.c. with MR766 was cross-neutralizing for PRVABC59, further suggesting that MR766 has not acquired mutations that alter ZIKV neutralization.

The ability of the MR766 ZIKV strain to elicit disease in the CD-1/ICR mouse model was dependent on the i.c. route of inoculation. CD-1/ICR mice inoculated by the i.p. route demonstrated no apparent disease progression after inoculation with either the MR766 or PRVABC59 strains, thus indicating the increased neurovirulence of the MR766 with an apparent lack of neuroinvasive properties of both viruses in immunocompetent mice. Although the extensive i.c. passaging of the MR766 virus has been proposed as the basis for its relatively high neurovirulence phenotype and crucial for the applicability of this model, the low-passage DakAr41524 strain elicited 30% mortality. It is also noteworthy that a low-passage African ZIKV isolate from the Central African Republic has demonstrated higher growth rates in neural stem cells and astrocytes compared with a contemporary Asian lineage strain from French Polynesia isolated in 2013.^27^ In addition, a study reported in 1956 using a low passage of the MR766 prototype ZIKV strain for intracerebral inoculation of adult mice (> 5 wko) resulted in symptoms of encephalitis within 6 days.^12^ These findings indicate that the neurovirulence differences observed here could be partially associated with inherent viral neurovirulence determinants of African genotype viruses and partially related to the extensive passaging of the MR766 virus in suckling mouse brain. Whereas PRVABC59 did not show neurovirulence in immunocompetent mice, closely related Asian genotype strains have caused severe neurological complications in humans. Other studies have suggested that this may be due to the interferon response, which can be antagonized by ZIKV in humans but not in mice.^28,29^

The development of an immunocompetent mouse model for ZIKV challenge is critical to future vaccine efficacy studies to assess the relative role of the interferon response of live attenuated and replication competent vaccine candidates. Intracranial inoculation of outbred mice with ZIKV strain MR766 provides an age-independent model for testing immunogenicity and protection of ZIKV vaccines.
